# Quantitative Optical Redox Imaging of Melanoma Xenografts with Different Metastatic Potentials

**DOI:** 10.3390/cancers16091669

**Published:** 2024-04-25

**Authors:** April Peng, He N. Xu, Lily Moon, Paul Zhang, Lin Z. Li

**Affiliations:** 1 Britton Chance Laboratory of Redox Imaging, Department of Radiology, Perelman School of Medicine, University of Pennsylvania, Philadelphia, PA 19104, USA; peng.april1@gmail.com (A.P.); hexu2@pennmedicine.upenn.edu (H.N.X.); lilymoon88@yahoo.com (L.M.); 2 Department of Bioengineering, School of Engineering and Applied Science, University of Pennsylvania, Philadelphia, PA 19104, USA; 3 Abramson Cancer Center, University of Pennsylvania, Philadelphia, PA 19104, USA; 4 Institute of Translational Medicine and Therapeutics, University of Pennsylvania, Philadelphia, PA 19104, USA; 5 Department of Pathology, University of Pennsylvania, Philadelphia, PA 19104, USA; paul.zhang2@pennmedicine.upenn.edu

**Keywords:** NADH, flavoprotein FAD, fluorescence, cancer aggressiveness, invasion, redox state

## Abstract

**Simple Summary:**

Use of cancer biomarkers for tumor aggressiveness is an unmet clinical need. Differentiation of high-risk versus low-risk tumors may guide physicians to select appropriate treatment strategies tailored to the risk level of individual patients. This study aimed to evaluate the value of an optical redox imaging technique for differentiating a human melanoma mouse xenograft model with a high risk of metastasis from a low-risk mouse model. Several imaging indices were found to be significantly different between the two models. The high-risk model was found to be in a more oxidized status and to have a higher intratumor redox heterogeneity. These findings may inform further research development of the optical redox imaging approach into translatable cancer biomarkers in the future.

**Abstract:**

To develop imaging biomarkers for tumors aggressiveness, our previous optical redox imaging (ORI) studies of the reduced nicotinamide adenine dinucleotide (NADH) and oxidized flavoproteins (Fp, containing flavin adenine dinucleotide, i.e., FAD) in tumor xenografts of human melanoma associated the high optical redox ratio (ORR = Fp/(Fp + NADH)) and its heterogeneity to the high invasive/metastatic potential, without having reported quantitative results for NADH and Fp. Here, we implemented a calibration procedure to facilitate imaging the nominal concentrations of tissue NADH and Fp in the mouse xenografts of two human melanoma lines, an indolent less metastatic A375P and a more metastatic C8161. Images of the redox indices (NADH, Fp, ORR) revealed the existence of more oxidized areas (OAs) and more reduced areas (RAs) within individual tumors. ORR was found to be higher and NADH lower in C8161 compared to that of A375P xenografts, both globally for the whole tumors and locally in OAs. The ORR in the OA can differentiate xenografts with a higher statistical significance than the global averaged ORR. H&E staining of the tumors indicated that the redox differences we identified were more likely due to intrinsically different cell metabolism, rather than variations in cell density.

## 1. Introduction

Effective prognostic biomarkers are needed in the clinic to accurately predict the risk of tumor progression to metastasis and facilitate personalized cancer treatment. Our long-term goal is to develop clinically translatable imaging biomarkers for tumor metastatic potential. The Chance redox scanner is a low temperature optical imaging system [1,2,3] that is applicable to tumor biopsies. It can determine the mitochondrial redox status of a tissue sample by measuring the fluorescence of reduced pyridine dinucleotides (NADH) and oxidized flavoproteins (Fp) containing flavin adenine dinucleotide (FAD) in the tissue. NADH and FAD are cofactors mediating important metabolic pathways including the Krebs cycle and oxidative phosphorylation in the mitochondrion. Previous studies demonstrated that these two intrinsic fluorescence signals and the optical redox ratios (ORR) are sensitive indicators of the mitochondrial metabolic states [4,5,6,7,8,9,10,11,12,13,14,15] and can differentiate between tumor and normal tissues and between tumor mouse models with different invasive/metastatic potentials or aggressiveness [16,17,18,19,20,21,22,23,24].

Particularly, using xenograft models of human melanoma, we have shown that the ORR of the oxidized regions of melanoma xenografts of five tumor lines correlate directly with the melanoma invasive potentials [20]. However, the reported redox ratios in the previous melanoma studies were based on relative fluorescence signal intensities and provided no quantifications of NADH and Fp concentrations. Later, we developed a calibration method [25] using external reference standards of NADH and FAD embedded adjacently to tissue to quantify the nominal concentrations of NADH and Fp and the concentration-based redox ratios. This calibration method allows the redox indices to be obtained that are less dependent on instrumental settings and quantitatively comparable across different imaging sessions. The method has been applied to studying breast cancer, colon cancer, lymphoma, and melanoma (under treatment) and as well as premalignant pancreatic tissues with the Chance redox scanner [17,18,21,23,24,26,27,28,29].

In the present study, our goals were twofold. First, we aimed to determine if the NADH and Fp nominal concentrations and concentration-based redox ratios can distinguish between the two melanoma xenografts with different metastatic potentials, i.e., the relatively indolent or less invasive/metastatic A375P cell line and the more invasive/metastatic C8161 cell line [20]. Second, we aimed to understand better the histological basis for the redox differences by analyzing the corresponding H&E staining images of these tumors. Our histological analysis was consistent with the metabolic intratumor heterogeneity for all redox indices (NADH, Fp and redox ratios) and supports the potential use of the optical redox imaging indices as possible biomarkers for melanoma metastatic potential in future clinical practice.

## 2. Materials and Methods

### 2.1. Mouse Xenograft Models and Sample Preparation

The C8161 cell line was originally established from the abdominal-wall metastasis of a female patient with recurrent malignant metastatic melanoma [30] and the A375P line was from a primary malignant melanoma in a female patient [31]. The Boyden chamber measurement indicated that C8161 has a significantly higher invasive potential than A375P [20,32]. The C8161 line was also highly metastatic in subcutaneously implanted mouse xenograft models by counting the number of spontaneous lung metastases in the mice, and the A375P line was barely metastatic or indolent [32,33].

In this study, the melanoma cells A375P and C8161 were cultured in a RPMI 1640 medium supplemented with 10% Fetal Bovine Serum and 20 mM HEPES solution [20]. Approximately 2 million melanoma cells from each line were subcutaneously injected into the hind leg of 7–9-week-old male athymic nude mice (NCI NCr-nu/nu; 20–35 g) acquired from the US National Cancer Institute. Once the size of each tumor reached around 15 mm, the host mice were anesthetized with ketamine/acepromazine (100/10 mg/Kg) and snap frozen in liquid nitrogen to retain the in vivo metabolic states of the tumors. On a dry ice-chilled metal plate, the tumors were carefully excised from the frozen host using a handsaw. The animal study protocol was approved by the Institutional Animal Care and Use Committee at the University of Pennsylvania (protocol # 804072).

Each frozen tumor specimen was mounted into a plastic cap using chilled mounting buffer (ethanol, glycerol, and water in a 1:3:6 ratio). Frozen FAD and NADH reference standards (plastic tubes containing 100 µM FAD or 100 µM NADH in 10 mM Tris-HCL buffer, pH~7) [21] were placed adjacent to the tissue sample, and then the entire sample cap was stored in liquid nitrogen, awaiting scanning.

### 2.2. Redox Scanning

Five tumors were imaged by redox scanning for each xenograft model. To initiate the scanning process, a tumor sample was placed into the liquid nitrogen chamber of the Chance redox scanner and milled until a flat cross section of the tumor was exposed. Redox scanning was conducted in darkness using a bifurcated fiber optic probe situated 70 µm above the surface of the tumor, which was completely submerged in liquid nitrogen. The light from a Mercury lamp was used to excite the tissue through a central optical fiber (200 µm in diameter) in the optical probe and the surrounding fibers collected the tissue fluorescence emission [34]. The Fp channel excitation filter was 430 ± 25 nm and the emission filter was 525 ± 32 nm. The NADH channel excitation filter was 360 ± 13 nm and the emissions filter was 430 ± 25 nm. To avoid signal saturation, neutral density filters of varying optical densities were placed in the emissions channels whenever necessary. A photomultiplier tube (R928 from Hamamatsu, Inc., Bridgewater, NJ, USA) collected the NADH and Fp emission signals, which were converted to fluorescence images by a computer. The scanning matrix for the tumors was either 64 × 128 or 128 × 128 with a resolution of 200 µm. Each tumor was scanned three times at various depths (1200 to 3600 µm) from sample surface to produce a total of 15 fluorescence images of the five tumors of the A375P line and 14 images for the five tumors of the C8161 line. One of the C8161 tumors only had two section scans.

### 2.3. Data Analysis

Using a customized program of MATLAB^®^ (R2016b, MathWorks, Natick, MA, USA), the background signal of a fluorescence image was removed by subtracting the average signal from a tissue-free region of interest (ROI) surrounding the tumor sample. The image was further processed by thresholding at a signal-to-noise (SNR) ratio of 7.5 (SNR of 3 or 4 were used for 4 out of 29 analyzed images per the discretion of the operator due to the relatively low SNR). The nominal NADH and Fp concentrations were calculated by comparing background-corrected signal intensities of the tumor tissue to those of the reference standards, and then the redox ratios (Fp/(Fp + NADH) and NADH/Fp) were quantified, pixel by pixel, based on the nominal concentrations of NADH and Fp. NADH/Fp was calculated to compare to the power of Fp/(Fp + NADH) in differentiating tumor models. For each image, a corresponding histogram was created for each redox index (Fp, NADH, and the redox ratios).

**Figure 1:**
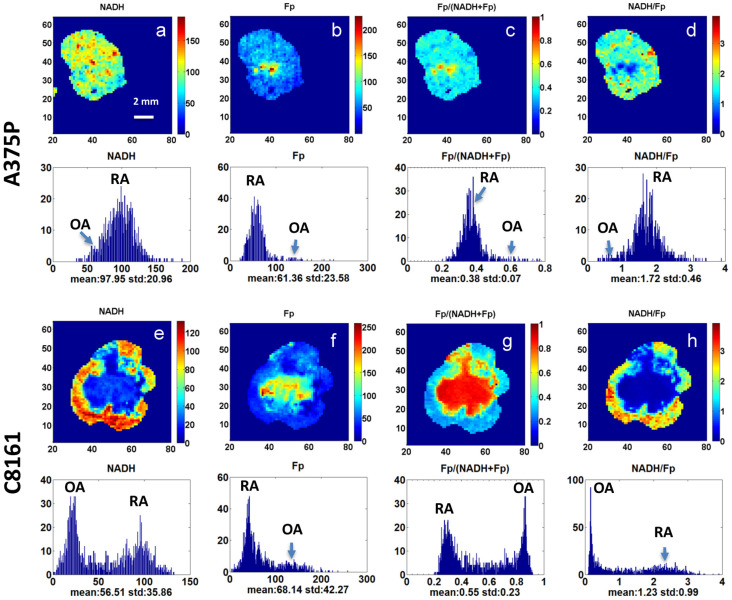
Typical redox images and corresponding histograms of A375P (**a**–**d**) and C8161 (**e**–**h**) tumors. OA: oxidized area. RA: reduced area. Color bars on the right side of images indicate the range of Fp or NADH signals (in µM) and redox ratios (no units). The numbers on the bottom and left sides of each image indicate the x and y coordinates, respectively. Pixel size 200 µm.

As shown by the representative redox images and corresponding histograms in Figure 1, significant intratumor heterogeneities were observed in both A375P and C8161 tumor models. Individual tumors may contain some relatively more oxidized areas (OAs, hotspots) with higher Fp and Fp/(Fp + NADH) and lower NADH and NADH/Fp ratio and some relatively more reduced areas (RAs) with lower Fp and Fp/(Fp + NADH) and higher NADH and NADH/Fp ratio. The A375P tumor cross-sectional areas were usually composed of large RA tissue areas and small OAs (hotspots). While the OAs of aggressive C8161 tumors were typically located in or nearby the geometrical center of a given cross section, surrounded by the RA in the periphery, hotspots in more indolent A375P tumors are more sporadic and not usually confined to the center of the cross section.

We determined the mean redox indices in the OA and RA regions of each tumor section in addition to the means of the whole cross section. For less heterogeneous A375P cross sections, only one main peak is apparent in the redox ratio histogram with a right-side tail (Figure 1c). The RA redox ratio values were visually estimated based on the position of the main peak while the OA redox ratio values were estimated based on the position of the right-side tail corresponding to the sporadic “hotspot” areas in the image. In C8161 cross sections with high heterogeneity, binomial peaks appear on the Fp/(Fp + NADH) redox ratio histograms (Figure 1g). The position of the less oxidized peak corresponds to the RA of the cross section, and the more oxidized peak corresponds to the OA of the cross section. Similarly, we analyzed the OA and RA regions for the Fp and NADH and NADH/Fp indices using visual readings of the histograms (Figure 1). Gaussian curves were also fitted to the redox ratio histograms of each slice and generated analysis results consistent with the findings of the OA and RA visual readings (Appendix A). The average redox indices of a whole image section or in its OA and RA were obtained and then averaged across all sections of each tumor. The mean values for each line were obtained by averaging across all tumors of each line.

### 2.4. H&E Staining and Cell Counting

H&E staining was performed to determine whether cell densities were different between tumor OAs and RAs. Three of the C8161 tumors and two of the A375P tumors were sliced and stained for histological comparisons. Four consecutive 12 µm-thick sections of each tumor, with the first section cut directly below the last section that was redox scanned, were stained with hematoxylin and eosin and scanned into high resolution images using Aperio ImageScope software (version 12.3.3). Next, we quantified the cell densities in randomly selected sample regions using the ImageJ program (FIJI version 2015 using Java 6) and verified the accuracy of the program by comparison with direct manual cell counts (Appendix B).

To compute the cell densities in the OA and RA regions of the tumors, we first manually oriented the stained images as closely as possible to match their corresponding redox images. We then randomly selected five OA and five RA sample regions, each with dimensions of 1100 × 1300 pixels, from tumors with relatively uniform H&E staining. For tumors with noticeably different histological regions, we selected five sample regions that best represented the different regions.

### 2.5. Statistical Analysis

Two-tailed Student’s *t* tests of unequal variance were performed to determine if significant differences in redox indices existed (*p* < 0.05 without the correction for the number of multiple comparisons). Paired two-tailed *t* tests were performed to test the statistical difference between the OA and RA of each tumor line. Cohen’s effect size *d* was calculated for a specific index by taking the index difference between two groups for comparison and then dividing it by the root mean square of the two group standard deviations (SDs).

## 3. Results

**Figure 2:**
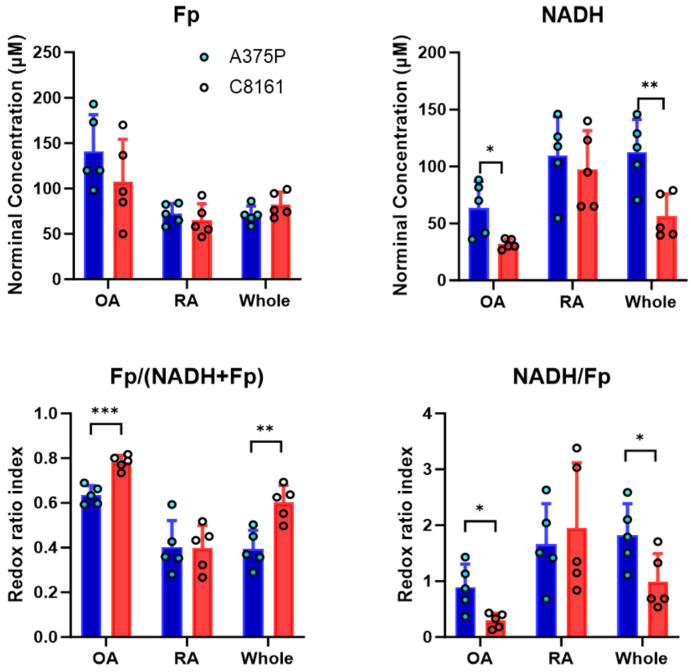
Comparison of the mean redox indices between A375P and C8161 tumors for tumor oxidized areas (OAs), reduced areas (RAs) and whole tumors. Circles represent individual tumors. * *p* < 0.05, ** *p* < 0.01,*** *p* < 0.001. Mean ± SD.

The subregion and whole-tumor averages of redox indices were plotted in Figure 2 for xenografts of the two tumor lines. For the whole-tumor indices, the NADH concentrations are significantly lower in C8161 tumors than in A375P tumors (*p* = 0.008), whereas Fp concentrations are similar between the two lines (*p* = 0.17). The average Fp/(Fp + NADH) of C8161 tumors is significantly higher than that of A375P tumors (*p* = 0.004). Accordingly, the average NADH/Fp of C8161 tumors is significantly lower than that of A375P tumors (*p* = 0.040). These findings indicate that, overall, the more aggressive tumors are more oxidized than the less aggressive tumors. The significant differences observed between NADH concentration, Fp/(Fp + NADH) ratio, and NADH/Fp ratio suggest that these redox parameters can distinguish between the two tumor lines of differing metastatic potentials.

Observing distinct patterns of intratumor heterogeneity (Figure 1), we further found significant or borderline significant differences between the redox indices of the OAs and RAs of each respective line, except for Fp concentration of the C8161 tumor line (Table 1). These results confirmed that the redox states of the OA and RA of each tumor line were significantly different. For both tumor lines, OA had higher Fp, lower NADH, higher Fp/(Fp + NADH), and lower NADH/Fp than RA. The percent differences for all group mean redox parameters between the C8161 OA and RA ((OA − RA)/RA%), except Fp concentration, exceed that of A375P tumors, indicating that the C8161 tumors exhibit higher heterogeneity than the A375P tumors. Furthermore, the *t* test of individual tumor percent differences showed that only the NADH percent difference is significantly different between the two tumor models (*p* = 0.03).

To determine whether these hotspots or OAs contributed significantly to the overall average redox indices of the A375P tumors, we compared the redox parameters of the A375P RA to the overall values of the A375P line. No significant differences were found in any of the redox indices (*p* > 0.5). This result supports our observation that the majority of A375P tumors are comprised of RA tissues. In other words, the presence of hotspots on A375P tumors does not significantly affect their overall redox status. The significant differences observed between the C8161 OA and whole-tumor indices including NADH (*p* = 0.024) and Fp/(Fp + NADH) (*p* = 0.005) indicate that the OAs are large enough to contribute significantly to the overall redox parameters, unlike the hotspots found in A375P tumors.

We then compare the OA or RA redox indices between the two tumor models in Figure 2. Comparison of the OA mean redox indices revealed significant differences in the NADH concentration, Fp/(Fp + NADH) and NADH/Fp of both lines (*p* = 0.039, *p* = 0.0003 and *p* = 0.029, respectively). Conversely, the Fp concentrations of the OAs showed no significant differences between the two tumor lines (*p* = 0.26). Between RAs of both lines, no significant differences were found in any of the four redox indices. Furthermore, when the overall redox parameters of A375P are compared to those of the C8161 RAs, no significant differences are detected for any redox indices.

In contrast, when the redox parameters of the C8161 OA were compared to the overall redox parameters of the A375P line, significant differences were found in the NADH concentration (*p* = 0.003), Fp/(Fp + NADH) (*p* = 0.0002), and NADH/Fp (*p* = 0.003), but not in Fp (*p* = 0.15). This comparison has the smallest *p* value and largest effect size *d* for the former three indices than the OA vs. OA comparison or the whole tumor comparison (Table 2). For Fp/(Fp + NADH), OA vs. OA comparison also has much smaller *p* and larger *d* than the whole tumor comparison. These findings indicate that the redox indices of tumor OAs can be used to distinguish tumor metastatic potential more effectively than whole-tumor averages with larger effect sizes and higher significance levels.

**Figure 3:**
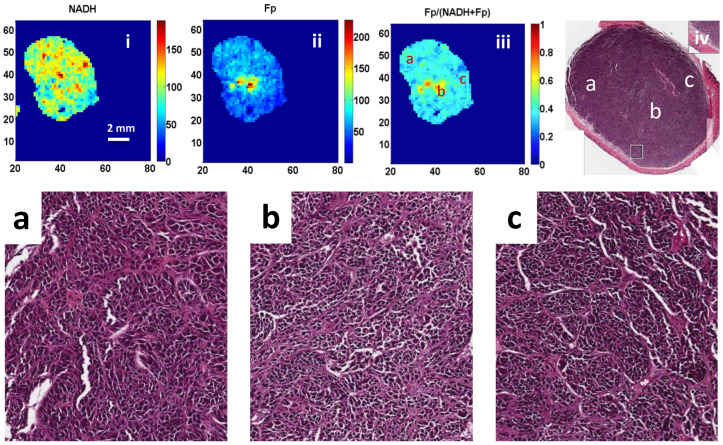
Comparison of the H&E stain photo of an A375P tumor slice with its corresponding Fp/(Fp + NADH) redox image. Panels (**i**–**iii**) show the NADH, Fp, and Fp/(Fp + NADH) images of a A375P tumor, respectively. Panel (**iv**) is the H&E image of the section right next to the redox image section. Panels (**a**–**c**) show blown-ups of H&E images corresponding to the locations marked in panels (**iii**,**iv**).

**Figure 4:**
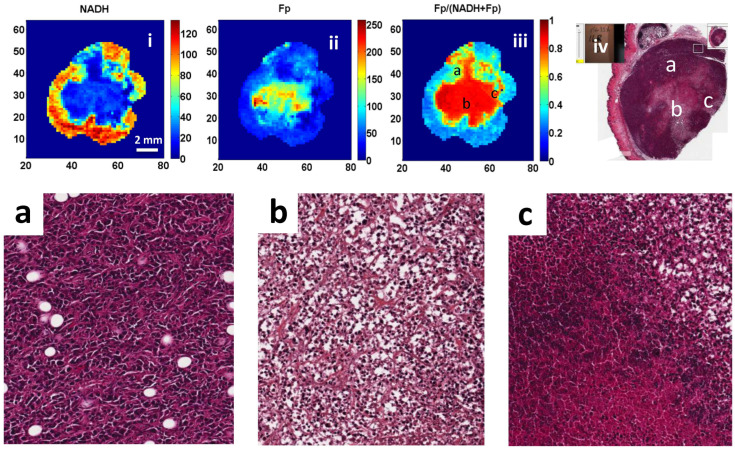
Comparison of the H&E stain photo of a C8161 tumor slice with its corresponding Fp/(Fp + NADH) redox image. Panels (**i**–**iii**) show the NADH, Fp, and Fp/(Fp + NADH) images of a C8161 tumor, respectively. Panel (**iv**) is the H&E image of the section right next to the redox image section. Panels (**a**–**c**) show blown-ups of H&E images corresponding to the locations marked in panels (**iii**,**iv**).

Figure 3 and Figure 4 show representative pictures of H&E staining of one A375P slice and one C8161 slice next to their corresponding redox images. Tumor cell density of the C8161 slice was more heterogeneous, whereas that of the A375P slice was relatively more homogeneous. In the OA of the C8161 staining, areas of degenerating cells and cellular lysis were found by a pathologist as evidenced by nuclear condensation and shrinkage as well as cytoplasmic clearing. In contrast, overall viable cells were found in the corresponding RA. Well preserved and viable cells were found throughout both the OA and RA of the A375P sections.

Five regions of interest (ROI) of 1300 × 1100 pixels were randomly selected from either the OA or the RA of each tumor and the cell number of each ROI was counted. Based on these data, we compared the cell density difference between OA and RA of individual tumors. Only one C8161 tumor showed significant differences (*p* = 0.01), and one of the three A375P tumors showed marginally significant differences between cell densities in OA and RA (*p* = 0.080). The cell numbers were also averaged across all ROIs to represent the mean OA or RA cell density of individual tumors. Comparison of the OAs and RAs of three A375P tumors showed no significant differences in the mean cell density (*p* = 0.66), and the same results were found for the C8161 tumors (*p* = 0.74) (Table 3). Moreover, no significant differences were found when comparing the number of cells between the OAs of the A375P and C8161 tumors (*p* = 0.77) as well as between the RAs of the two tumor lines (*p* = 0.20).

## 4. Discussion

Our previous ORI studies of human melanoma xenografts reported that melanoma metastatic risk positively correlates with the optical redox ratio Fp/(Fp + NADH) of OAs, where neither NADH nor Fp was quantified [19,20]. Later, we developed a calibration procedure utilizing reference standards so that the nominal tissue concentrations of NADH and Fp can be quantitatively obtained and compared across imaging sessions and tissue samples. We showed that in addition to the redox ratios, both NADH and Fp nominal concentrations were found to be significantly higher in breast tumors than adjacent normal tissue [18]. Furthermore, the NADH or Fp nominal concentrations can differentiate between breast and colon cancer xenografts of different metastatic potentials [21,26]. NADH was found to be significantly lower in more aggressive colon cancer xenografts while Fp was found to be significantly lower in more aggressive breast cancer xenografts.

The present work is the first quantitative ORI study of melanoma xenografts with different aggressiveness utilizing reference standards so that both Fp and NADH were quantified along with the redox ratios. We also performed a histological analysis of cell densities in the tumor OA and RA and compared the cell density difference between OA and RA and between models. By doing so, we obtained biochemical results that were consistent with previous studies about the metabolic state in human melanoma xenografts.

Similar to the previous studies, this study showed that in general, the more invasive and metastatic C8161 tumors are more oxidized than the less invasive and metastatic A375P tumors. On average, C8161 tumors displayed higher Fp/(Fp + NADH) ratios and lower NADH/Fp ratios than the indolent A375P tumors. In addition, the metastatic tumors also had lower globally averaged NADH. No significant difference was found between the mean Fp concentrations of the two tumor lines. This study further confirmed the previously known fact that both melanoma models exhibited oxidized and reduced areas. Despite the presence of OA areas in A375P tumors, the C8161 tumors had more distinct oxidized areas and were thus more heterogeneous. The more metastatic tumors are generally associated with higher Fp/(Fp + NADH) ratios and intratumor heterogeneities as we also observed in human breast and colon tumor in mouse xenografts [21,26].

To further characterize the intratumor redox heterogeneity, we utilized the histogram OA–RA analysis to extract the redox indices within both OAs and RAs. The OA–RA analysis of redox ratio histograms by the Gaussian fit were consistent with the results obtained by visual readings directly from the histograms. Both tumor models showed significantly lower NADH and higher Fp/(Fp + NADH) ratios in OAs compared to RAs (Table 1). However, for both indices, greater percentage differences between OA and RA were identified in C8161 tumors than in A375P tumors, indicating a higher intratumor heterogeneity. Comparison of redox indices between the OAs of both tumor lines indicated that Fp/(Fp + NADH) is more sensitive to differences between the two tumor lines than NADH/Fp, as it provided a smaller *p*-value and larger effect size (Table 2). Both redox ratios in OAs can differentiate the two tumor models more effectively than the whole-tumor averaged redox ratios. The result of more oxidized OAs in the more metastatic tumors is consistent with our previous OA–RA analysis of the Fp/(Fp + NADH) ratios in human colon cancer xenografts [26].

Furthermore, NADH concentrations were also found to be significantly different between the OAs of both tumor lines (*p* = 0.039). Conversely, the OA Fp concentration did not appear to significantly distinguish the two tumor lines. No significant differences in any of the redox indices were found between the RAs of tumor lines. In addition, comparison of the A375P whole-tumor redox indices to the RA redox indices found no significant differences. These results indicate that OAs or hotspots do not occupy large enough areas (~1 mm^2^ or less) to contribute significantly to the overall redox states of A375P tumors. Therefore, indolent tumors may be distinguished from more aggressive tumors simply by comparing their overall redox state to the OAs of aggressive tumors, which produced more significant *p*-values and larger effect sizes (Table 2). Based on the effect size that we calculated for all the redox indices, the OA Fp/(Fp + NADH) was found to be the most sensitive indicator of metastatic potential difference.

**Figure A2:**
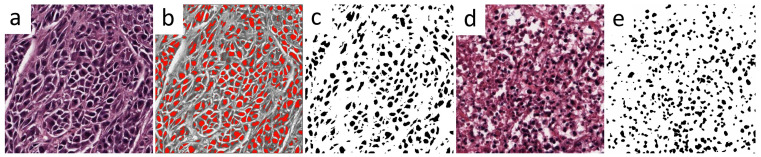
ImageJ software produced cell counting in 500 × 500 pixel sample areas of representative tumor sections. (**a**–**c**) A375P: H&E image (**a**); red channel image after ImageJ split (**b**); final black-white image for cell counting (**c**). (**d**,**e**) C8161 oxidized area: H&E image (**d**); black–white image for cell counting (**e**).

Although fluorescence signals are expected to be proportional to cell density, we did not find a significant difference in cell densities between the OA and RA within each tumor line and between tumor lines. These results imply that the significantly different ORI indices between OA and RA and between tumor models are due to the intrinsic biological differences (e.g., cell phenotypes or genotypes) rather than cell density differences. Previously, it was proposed that the OAs of metastatic melanomas were under substrate starvation and the harsh microenvironment in the OAs generated more oxidized redox states, which correlated with metastatic potential [20]. Histological staining demonstrated the existence of a large amount of viable cells with intact nuclei in the OA areas of tumors (Figure 4 and Figure A2) consistent with our previous histological and TUNEL analysis of melanoma xenografts [35]. Histological H&E staining performed in this study found significant differences in the cell morphologies between the OAs and RAs of C8161 tumors, but not in A375P tumors. However, ORI demonstrated significant metabolic differences between OAs and RAs for both tumor models (Table 1).

Therefore, optical redox imaging provides valuable metabolic information and can be used in conjunction with H&E staining to provide a more complete view of tumors. Further studies are needed to explore the exact mechanism underlying the redox imaging differences between the metastatic and indolent tumors. In general, our studies of mouse xenografts of human melanoma, breast cancer, and colon cancer cell lines with different metastatic potential indicate that the more metastatic tumors are characterized by an oxidized intratumor subpopulation with lower NADH and higher Fp/(Fp + NADH) correlated with the aggressiveness, likely implying higher oxidative stress and more oxidized redox status. ROS was shown to decrease NADH and increase Fp/(Fp + NADH) in a dose-dependent manner in cancer cells [9]. It is also likely that lower NADH and higher redox ratio can be due to more consumption of NADH by a higher rate of mitochondrial electron transport and ATP synthesis, or less generation of NADH due to less entry of pyruvate into the TCA cycle (under enhanced aerobic glycolysis or Warburg effect). The exact mechanism remains to be investigated.

Although the push for proactive melanoma screenings and vigilance has dramatically increased the rate of early detection and decreased the overall mortality rate, it remains a challenge to differentiate indolent melanoma from clinically more relevant melanomas [36]. Technologies need to be developed to aid clinical diagnoses and minimize overdiagnosis and overtreatment. The Chance redox scanner has been applied to imaging the metabolic heterogeneity of clinical biopsy samples [16,17,18] that may assist in clinical cancer management. In addition, multiphoton imaging of NADH and Fp can be integrated with other nonlinear optical measurements to study tissue metabolism, function, and structures in vivo [15,37]. As this work and previous studies suggest, optical redox imaging indices may potentially differentiate indolent and metastatic tumors in the clinic, and they warrant further validation and development.

We acknowledge there are several limitations of this study that we may address in the future. First, since tumor xenografts commonly have inter-tumor variations depending on the animal host and local environment in the site of implantation, our sample size of five xenografts for each cancer line may be insufficient despite statistical significance being achieved. We may increase the sample size and study more tumor lines with intermediate levels of invasive/metastatic potential to confirm the results. Second, xenografts in athymic nude mice are tumor progression models with suppressed immune system and lacking human immune cells. However, immune cells are present in human tumors. To translate the redox indices biomarkers into the clinic, we need to understand how immune cells may affect the redox status and invasive/metastatic potential of cancer cells. It is likely that the intratumor immune cells will modulate the tumor cell redox status in human tumors, presumably depending on the cell type and microenvironment. However, despite ORI having been utilized for imaging immune cells including cocultures with cancer cells [38,39,40,41,42], this remains to be investigated in the future. We could study cocultures of cancer and immune cells as well as syngeneic mouse tumor models to investigate the connection between ORI redox indices and tumor invasive/metastatic potentials in the presence of immune cells.

## 5. Conclusions

Utilizing the internal reference standards of NADH and FAD, this study indicates that the globally averaged NADH concentration and redox ratio but not Fp concentration can differentiate the two melanoma xenografts (indolent A375P and metastatic C8161). Having observed significant intratumor redox heterogeneities or redox subpopulations in these melanoma xenografts, we performed the intratumor subpopulation (OA and RA) analysis and identified significant differences of all redox indices except Fp for C8161 between OA and RA for both melanoma models. While no significant redox differences were found between the RAs of the two models, NADH and the redox ratios in OAs can differentiate the more metastatic model from the less metastatic one. Furthermore, the redox ratio Fp/(Fp + NADH) in OAs can differentiate between the two tumor models with the highest statistical significance and largest effect size than other indices in OAs and all the globally averaged indices. Our histological analysis of tumors found same or similar cell densities between OA and RA and between melanoma models, supporting the hypothesis that the observed redox differences are due to altered cell metabolism.

## Figures and Tables

**Table 1:** Comparison of mean redox indices between OA and RA of each tumor line (*n* = 5).

Redox Indices	Percent Difference of A375P ^#^ (%)	*p* *	Percent Difference of C8161 (%)	*p*
NADH (µM)	−42.0	0.012	−67.4	0.008
Fp (µM)	94.9	0.011	65.1	0.154
Fp/(Fp + NADH)	57.8	0.005	96.4	0.001
NADH/FP	−46.1	0.066	−84.8	0.046

^#^ Calculated as (OA − RA)/RA using group means. * Student’s *t* test *p* value comparing the redox indices between the OAs and RAs of each tumor line.

**Table 2:** Comparison of the significance levels and effect sizes for redox indices differentiating two tumor lines (*n* = 5).

Redox Indices	C8161 Whole vs. A375P Whole		C8161 OA vs. A375P OA		C8161 OA vs. A375P Whole	
	*p* ^#^	*d* *	*p*	*d*	*p*	*d*
NADH (µM)	0.008	−2.30	0.039	−1.87	0.003	−3.95
Fp/(Fp + NADH)	0.004	2.58	0.0003	3.96	0.0002	6.15
NADH/FP	0.040	−1.55	0.029	−1.95	0.003	−3.70

^#^ Student’s *t* test significance level; * Cohn’s effect size.

**Table 3:** Comparison of OA and RA cell densities of the C8161 and A375P tumor lines (mean ± SD).

Tumor Cell Density	OA	RA	*p* ^#^
A375P (ROI *n* = 15)	2221 ± 126	2152 ± 48.7	0.66
C8161 (ROI *n* = 15)	2294 ± 358	2376 ± 169	0.74
*p* *	0.77	0.20	

^#^ Comparing OA versus RA. * Comparing tumor models.

**Figure A1:**
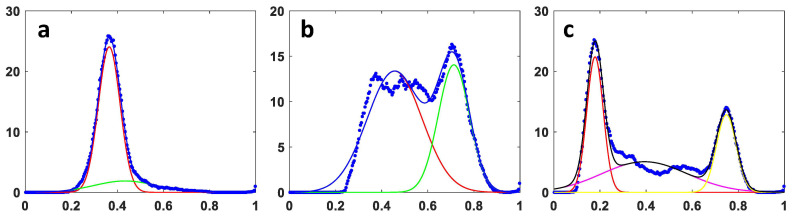
Gaussian fits of representative Fp/(Fp + NADH) histograms from tumor sections containing OA and RA. (**a**) A375P; (**b**,**c**) C8161 fit with two to three Gaussian curves. Dotted lines represent the histograms. The red, green, magenta, and yellow lines are individual Gaussian function curves used for fitting, and the blue (**a**,**b**) or black (**c**) lines represent the sum of individual Gaussian curves fitting the histograms. RA: red, OA: green (**a**,**b**) or yellow (**c**), intermediate areas: magenta.

## Data Availability

Raw data will be available for sharing upon request and mutual agreement.

## Appendix A. Gaussian Fitting of Fp/(Fp + NADH) Histograms and Comparison to Visual Resulting Analysis

We fitted representative Fp/(Fp + NADH) histograms with several Gaussian curves to extract the redox ratios for the tumor reduced areas (RAs) and oxidized areas (OAs). All histograms were smoothed by taking a moving average across 19 neighboring points (smooth index = 19) before the Gaussian fitting. Figure A1a and b show the fitting of histograms from an A375P and a C8161 section, respectively, with two Gaussian curves, with the peak positions of Gaussian curves corresponding to the average redox ratios for RA and OA. The histograms of two C8161 tumors with six sections were so wide that three Gaussian functions were needed to fit them and extract the mean redox ratios for RA or OA subareas (Figure A1c). The overarching criteria for determining the best Gaussian fit method for each slice involved keeping the RMSE (root mean square error) values, which report goodness of fit. All histograms that were fitted with three Gaussian curves reported lower RMSE values than when fit with two Gaussian curves. One C8161 slice produced problematically high RMSE values despite its fit with 3 Gaussians. To remedy the issue, this slice was again fitted with four Gaussian curves resulting in an RMSE value of 1.03. All final-fit RMSE values were less than 1.1. Redox ratio indices for RA or OA were averaged across individual sections for each tumor and then averaged for all five tumors within each xenograft model.

Comparison of the analysis results from visual reading and Gaussian fitting of histograms found consistent results in Table A1. Overall, each of the redox ratios found through Gaussian curve fitting closely matched those obtained by histogram visual reading analysis. There are no significant differences between the results of both methods. Compared to the Fp/(Fp + NADH) peak values found for A375P OAs (hotspots) through Gaussian curve fitting, the peak values from direct visual analysis tend to be higher. This is presumably attributed to the difficulties in determining the exact location of an oxidized peak in A375P tumors, which only shows as a weak broad peak on the right side of the much larger reduced peak.

**Table A1:** Comparison of Fp/(Fp + NADH) redox values (mean ± SD) obtained through Gaussian curve fitting and visual reading analysis (*n* = 5).

Methods	A375P OA	C8161 OA	A375P RA	C8161 RA
OA/RA visual reading	0.635 ± 0.042	0.782 ± 0.031	0.403 ± 0.118	0.398 ± 0.101
Gaussian curve fitting	0.546 ± 0.184	0.788 ± 0.035	0.394 ± 0.129	0.447 ± 0.152
*p* ^#^	0.25	0.67	0.41	0.20

^#^ Paired *t* test comparing visual reading and Gaussian curve fitting.

Our analysis with the Gaussian curve fitting of Fp/(Fp + NADH) histograms found that the OA peaks of C8161 tumors were significantly more oxidized than those of the A375P tumors (*p* = 0.04). However, comparison of the average RA peak values of both lines showed no significant difference between both lines (*p* = 0.57). The average Fp/(Fp + NADH) percent of change between the RA and OA is 106% in C8161 and 39% in A375P, indicating that the RA and OA peaks were further apart in the C8161 tumors than in the A375P tumors. These results are consistent with the results from direct visual reading analysis (Table 1).

## Appendix B. Validation of Cell Counting on H&E Slides Using ImageJ Software

We compared the cell counts from ImageJ analysis with the direct visual cell counts for selected regions of 250 × 250 pixels to verify the accuracy of the ImageJ program (FIJI version 2015 using Java 6). To provide a uniform method of analysis, one OA and one RA were randomly selected from the third H&E section of each tumor. Unfiltered H&E sample images (Figure A2a) were processed using ImageJ software, where the images were split into their separate red, blue, and green color channels. The red channel image (Figure A2b) was chosen for analysis because it offered the largest grayscale contrast between the nuclei and the surrounding extracellular material. A grayscale threshold was then set to convert all nuclei into clusters of black pixels while everything outside the nuclei was converted to white pixels (Figure A2c). These black pixel clusters were counted by ImageJ. Using the Remove Outliers command of ImageJ, we were able to exclude the black pixel clusters under a preset diameter.

Same-image process for the oxidized areas in C8161 tumors is also demonstrated in Figure A2d,e. We acknowledge that if the oxidized areas are necrotic, extensive nuclear fragmentation can mislead the cell counting results. However, although the cell morphology in the oxidized areas of C8161 tumors was very different from that in the reduced area, the oxidized areas of C8161 tumors exhibited a vascular-like network with mostly intact nuclei indicating non-apoptotic cells [35] (see Figure A2d). Thus, we could count the cell densities even for the OA areas of C8161.

In total we analyzed ten 250 × 250 pixel regions, five taken from tumor OA and another five from tumor RA. We revealed no significant differences between ImageJ cell counts and direct visual cell counts (Table A2). Furthermore, a linear correlation test was performed between the direct visual counting results and the ImageJ cell counts. The linear correlation coefficient was r = 0.91 (*p* = 0.0002), indicating a strong, positive linear relationship between the two methods of cell counting. Therefore, we found ImageJ to be a reliable means of counting the number of cells in H&E staining sections.

**Table A2:** Cell counts (mean ± SD) from visual counting and ImageJ analysis.

Average ImageJ Cell Count (*n* = 10)	Average Visual Cell Count (*n* = 10)	*p*-Value
106 ± 17.3	108 ± 16.3	0.76
